# Cloning and Characterization of a Flavonol Synthase Gene from *Scutellaria baicalensis*

**DOI:** 10.1155/2014/980740

**Published:** 2014-01-28

**Authors:** Yeon Bok Kim, KwangSoo Kim, YeJi Kim, Pham Anh Tuan, Haeng Hoon Kim, Jin Woong Cho, Sang Un Park

**Affiliations:** ^1^Department of Crop Science, Chungnam National University, 99 Daehak-ro, Yuseong-gu, Daejeon 305-764, Republic of Korea; ^2^Department of Biochemistry and Molecular Biology, Baylor College of Medicine One Baylor Plaza, Houston, TX 77030, USA; ^3^Department of Well-Being Resources, Sunchon National University, 413 Jungangno, Suncheon, Jeollanam-do 540-742, Republic of Korea

## Abstract

Flavonols are the most abundant of all the flavonoids and play pivotal roles in a variety of plants. We isolated a cDNA clone encoding flavonol synthase from *Scutellaria baicalensis* (SbFLS). The SbFLS cDNA is 1011 bp long, encodes 336 amino acid residues, and belongs to a family of 2-oxoglutarate-dependent dioxygenases. The overall structure of *SbFLS* is very similar to that of *Arabidopsis thaliana* anthocyanidin synthase (AtANS), with a **β** jelly-roll fold surrounded by tens of short and long **α**-helices. *SbFLS* was constitutively expressed in the roots, stems, leaves, and flowers, with particularly high expression in the roots and flowers. SbFLS transcript levels in the roots were 376-, 70-, and 2.5-fold higher than in the leaves, stems, and flowers. The myricetin content was significantly higher than that of kaempferol and quercetin. Therefore, we suggest that SbFLS mediates flavonol formation in the different organs of *S. baicalensis*. Our study may contribute to the knowledge of the role of FLS in *S. baicalensis*.

## 1. Introduction

*Scutellaria baicalensis* Georgi (Lamiaceae) is one of the most popular herbs in several oriental countries, where it is used to treat inflammation, respiratory tract infections, diarrhea, dysentery, jaundice/liver disorders, hypertension, hemorrhaging, and insomnia [1]. *Scutellaria* is a mild relaxant that affects the neural and muscular-skeletal systems [2]. *S. baicalensis* root has abundant flavones, a class of plant flavonoids [3].

**Figure 1:**
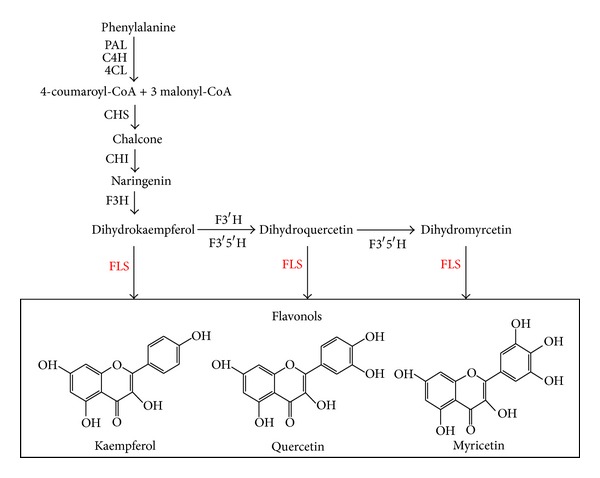
Flavonol biosynthesis in plants (redrawn from [37]). The red letter and black box indicate the enzyme and compound analyzed in this study. PAL, phenylalanine ammonia lyase; C4H, cinnamate 4-hydroxylase; 4CL, 4-coumaroyl CoA ligase; CHS, chalcone synthase; CHI, chalcone isomerase; F3H, flavone 3-hydroxylase; F3′H, flavonoid 3′-hydroxylase; F3′5′H, flavonoid 3′5′-hydroxylase; FLS, flavonol synthase.

Polyphenols, synthesized via the phenylpropanoid pathway, can be classified into (iso)flavonoids, coumarins, aurones, condensed tannins, and anthocyanins [4]. Flavonoids are important secondary metabolites derived from malonyl-CoA and the aromatic amino acid phenylalanine [5]; approximately 8000 different flavonoid compounds have been identified [6]. Flavonols are the most abundant of all the flavonoids [7] and play pivotal roles in a variety of plants [8]. Flavonols are particularly well known for their antioxidant, anti-inflammatory, antiangiogenic, antiproliferative, and neuropharmacological properties and thereby account for the health-promoting effects of grapes and many other fruits [8, 9]. Flavonols are synthesized from dihydrokaempferol (DHK), dihydroquercetin (DHQ), or dihydromyricetin (DHM) by flavonol synthase (FLS) enzymes (Figure 1). FLS belongs to a growing family of 2-oxoglutarate-dependent dioxygenase (2-ODD) nonheme ferrous enzymes that also includes the flavonoid enzymes flavanone-3*β*-hydroxylase (F3H), flavone synthase *Ι* (FS *Ι*), and leucoanthocyanidin reductase (LDOX) [10]. FLS activity was first characterized in irradiated parsley cells [11]. The first FLS gene was isolated from *Petunia hybrida* and expressed in yeast [12]. FLS genes have recently been cloned and characterized in *Populus tremula *[13], *Zea mays *[14], *Fagopyrum tataricum *[15], and *Ginkgo biloba *[16]. Cloning and characterization of FLS from *S. baicalensis *have not been reported.

In this study, a full-length cDNA encoding FLS was isolated from *S. baicalensis *by using next-generation sequencing platforms (NGS) (Roche 454 FLX+ and Illumina HiSeq 2000) (unpublished data). The mRNA transcript levels and flavonol accumulation from different organs of *S. baicalensis *were analyzed by real-time PCR and high-performance liquid chromatography (HPLC). The cloning and characterization of SbFLS may provide a foundation to elucidate the mechanism of flavonol synthesis in *S. baicalensis*.

## 2. Materials and Methods

### 2.1. Plant Material and Growth Conditions

The seeds of* S. baicalensis *were sown in May 2012, and then seedlings were transferred into pots filled with perlite-mixed soil. Seedlings were grown under 16 h light and 8 h dark conditions in the greenhouse (25°C and 50% humidity) at Chungnam National University (Daejeon, Korea). We used at least nine pots for biological repeats. Each organ (flowers, stems, leaves, and roots) was collected for two weeks after the first day of flowering. All samples were frozen in liquid nitrogen upon collection and stored at −80°C prior to RNA isolation and HPLC.

### 2.2. Cloning of the cDNA Encoding Flavonol Synthase

A putative flavonol synthase was obtained from *S. baicalensis* by next-generation sequencing (NGS) (Roche 454 FLX+ and Illumina HiSeq 2000) (unpublished data). We obtained 1,226,938 reads from Roche 454 FLX+ and 161,417,646 reads from Illumina HiSeq 2000. The *de novo* assembly of the 454 and Illumina high-quality reads (95% and 75%) were resulted more than 82 Mb sequence length with an average contig read length of 1,614 bp in 51,188 contigs which is the long length contigs with at least 500 bp. Among these contigs, a total of 39,581 contigs were annotated by BLASTX against the public NCBI nonredundant database. In particular, putative SbFLS sequence was obtained by BLASTX for comparison. The primers for the open reading frame (ORF) were as follows: forward, 5′-ATGGAGGTTGGGAGAGTG-3′; reverse, 5′-TCACTGGGGAAGCTTATTAAGCTTAC-3′. The TM Calculator program (http://bioinfo.ut.ee/primer3-0.4.0/) was used to compute the PCR annealing temperatures. The PCR program consisted of denaturation at 94°C for 1 min, annealing at 45°C for 1 min, and an extension at 72°C for 1 min. Thirty cycles were preceded by denaturation step at 94°C for 3 min, followed by a final extension at 72°C for 10 min. The amplified product was purified and cloned into the T-blunt vector (SolGent, Daejeon, Korea) and sequenced. The full-length FLS sequence was aligned using BioEdit Sequence Alignment Editor, version 5.0.9 [17]. For putative conserved domains, we used a GenBank conserved domain database search and function analysis service in NCBI.

### 2.3. Total RNA Extraction and Quantitative Real-Time RT-PCR

Total RNA was isolated from *S. baicalensis *organs with an RNeasy Plant Mini Kit (Qiagen, Valencia, CA, USA). First-strand cDNA was synthesized from total RNA (1 *μ*g) with ReverTra Ace-*α* - (Toyobo, Osaka, Japan) and oligo (dT)_20_ primer according to the manufacturer's protocol. ORF and gene-specific primers were designed using an online program (http://frodo.wi.mit.edu/primers3). The gene-specific primer sets were designed for SbFLS-RT (forward), 5′-ACCCAACGAGGTTCAAGGAC-3′, and SbFLS-RT (reverse), 5′-TGGTGGGGTCTCTTCATTCA-3′. Real-time PCR was performed in a 20 *μ*L reaction volume with 0.5 *μ*M each primer and 2× SYBR Green real-time PCR master mix (Toyobo, Osaka, Japan). Cycling conditions were as follows: 1 cycle of 95°C for 3 min, followed by 40 cycles of 95°C for 15 s, 72°C for 20 s, and annealing at 50°C. Real-time RT-PCR was carried out in triplicate on a CFX96 real-time PCR system (Bio-Rad; Hercules, CA, USA). The actin gene (GenBank accession number HQ847728) was used as an internal reference to standardize the cDNA template concentration.

### 2.4. Bioinformatic Analysis of Flavonol Synthase

The deduced amino acid sequence of SbFLS was aligned using the BioEdit Sequence Alignment Editor, version 5.0.9 (Department of Microbiology, North Carolina State University, Raleigh, NC, USA) [17]. Theoretical molecular weights (MW) and isoelectric points (pI) were calculated using the Compute pI/Mw tool (http://ca.expasy.org/tools/pi_tool.html). Putative target localization of SbFLS was predicted by using WoLF PSORT (http://psort.hgc.jp/form.html) [18]. The phylogenetic tree was constructed using an online program (http://www.phylogeny.fr/) [19]. Secondary structure prediction was investigated by network protein sequence analysis at SOPMA (http://npsa-pbil.ibcp.fr/cgi-bin/npsa_automat.pl?page=/NPSA/npsa_sopma.html) [20]. The three-dimensional (3D) structure was built using the SWISS-MODEL program and illustrated with the PyMOL viewer.

### 2.5. HPLC Analysis

Flavonol content was determined as described in the studies by Li et al. [15] and Kovács et al. [21], with a modification. Flavonol standards, kaempferol, quercetin, and myricetin were purchased from Sigma-Aldrich. The harvested organs (flowers, stems, leaves, and roots) were freeze-dried for 48 h and ground into a fine powder using a mortar and pestle. Powdered samples (100 mg) were extracted by sonication in 80% (v/v) methanol at room temperature for 60 min. During extraction, samples were vortexed every 15 min. After extraction, the extracts were centrifuged at 14,000 rpm for 10 min and the supernatant was filtered with a 0.45 *μ*m Acrodisc syringe filter (Pall Corp.; Port Washington, NY) for HPLC. HPLC was performed with a C18 column (250 × 4.6 mm, 5 *μ*m; RStech; Daejeon, Korea). The mobile phase was a gradient prepared from mixtures of methanol and 0.1% acetic acid. The flow rate was maintained at 0.7 mL/min. An injection volume of 20 *μ*L and wavelength of 270 nm were used for detection. The compounds in the sample were determined using a standard curve. Determinations were performed after 3 separate extractions of each sample and each extract was analyzed in triplicate (*n* = 3).

## 3. Results and Discussion

### 3.1. Cloning and Sequencing Analysis of FLS

**Figure 2:**
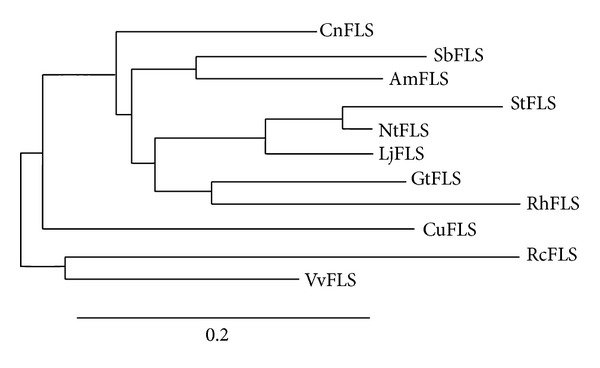
Phylogenetic relationships of FLS protein from *S. baicalensis* and selected species. GenBank accession numbers are as follows: NtFLS (*Nicotiana tabacum*, ABE28017), AmFLS (*Antirrhinum majus*, ABB53382), CnFLS (*Camellia nitidissima*, ADZ28516), StFLS (*Solanum tuberosum*, ACN81826), GtFLS (*Gentiana triflora*, BAK09226), VvFLS (*Vitis vinifera*, BAE75809), RhFLS (*Rudbeckia hirta*, ABN79672), CuFLS (*Citrus unshiu*, BAA36554), LjFLS (*Lonicera japonica*, AFJ44313), RcFLS (*Ricinus communis*, XP_002513772), and SbFLS (*S. baicalensis*, KC404852).

Based on *S. baicalensis *NGS data (unpublished), we designed primers specific to the open reading frame (ORF) and flower cDNA to generate a 1011 bp full-length fragment for use in PCR. The SbFLS amino acid sequence obtained from the PCR fragment contained 1 more residue than indicated by the NGS data (data not shown). SbFLS (GenBank accession no. KC404852) was composed of 336 amino acids with a MW and pI of 38.3 kDa and 5.51, respectively. This result corresponded with the published sequence from *Arabidopsis *[22]. *G. biloba* FLS has a 1023 bp ORF encoding a 340 amino acid protein, MW of 38.7 kDa, and pI of 5.75 [16]. The deduced SbFLS shared 77, 74, 72, 72, 71, and 70% identity with FLS proteins from *Antirrhinum majus *(ABB53382),* Nicotiana tabacum *(ABE28017),* Camellia nitidissima *(ADZ28516), *Gentiana triflora *(BAK09226), *Solanum tuberosum *(ACN81826), and* Vitis vinifera *(BAE75809), respectively (Supplementary Figure S1 available online at http://dx.doi.org/10.1155/2014/980740). Owens et al. [23] reported that the genome of *Arabidopsis thaliana *contains 5 sequences with high similarity to *AtFLS1*, a previously characterized flavonol synthase gene; 4 AtFLS isoforms are located on chromosome 5. A GenBank conserved domain database search and function analysis revealed that SbFLS has putative conserved domains belonging to the 20G-FeII_Oxy super family and the highly conserved N-terminal region of proteins with 2-oxoglutarate/Fe(II)-dependent dioxygenase activity. This is consistent with the results reported by Xu et al. [16]. Moreover, SbFLS had conserved sequence motifs, including the HXD motif [15] for ligating ferrous iron and the RXS motif for binding 2-oxoglutarate (2OG) [16]. To determine the relationship between the putative SbFLS protein and other plant FLSs, we performed phylogenetic analysis (Figure 2). The SbFLS phylogeny was clustered into 2 distinct groups and showed the closest relationship with *A. majus*. The subcellular targeting of SbFLS was predicted by PSORT to be nuclear and cytosolic. AtFLS1 localizes to the cytoplasm and nucleus [22], whereas maize FLS localizes in the ER and perinuclear region [24]. SOPMA [20] indicated that SbFLS contains 121 (36%) alpha-helices, 61 (18.6%) extended strands, 22 (6.6%) beta turns, and 132 (39.3%) random coils, respectively.

### 3.2. Modeled 3D-Structure of SbFLS

**Figure 3:**
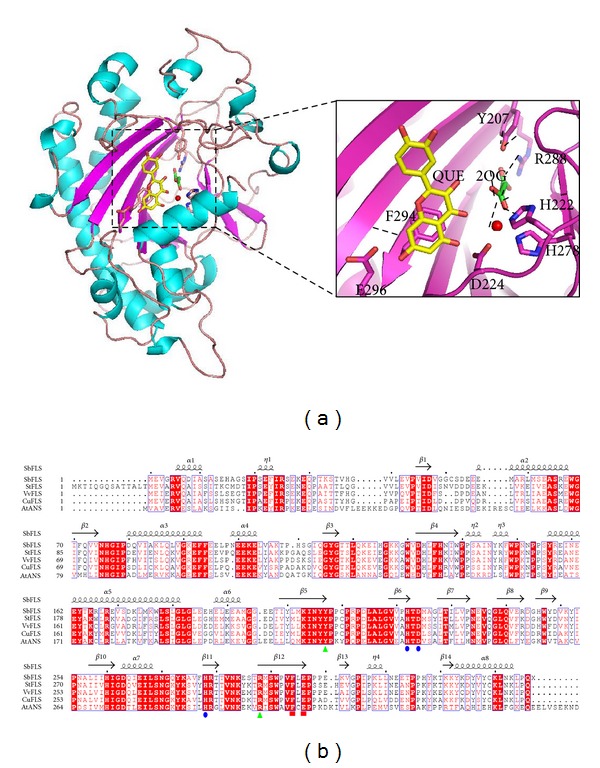
The structural model of SbFLS. (a) The overall and detailed views of the structure of SbFLS showing the *β* jelly-roll fold and presumed catalytic site are modeled using SWISS-MODEL server (http://swissmodel.expasy.org/) [27]. Iron atom is represented by a red dot. Substrates quercetin (QUE) and 2-oxoglutarate (2OG) are indicated in yellow and green. The iron, QUE, and 2OG are positioned by the same structure identified in the AtANS complex (PDB code 1GP6). Possible hydrogen bonds are indicated by dashed lines. (b) Sequence alignment of AtANS and various FLSs from *S. baicalensis *(SbFLS),* V. vinifera *(VvFLS),* C. unshiu *(CuFLS), and *A. thaliana* (AtFLS). The secondary structure was drawn from the modeled structure of SbFLS. Residues coordinating with an iron atom are represented by blue ovals. Green triangles and red rectangles indicate residues that interact with 2OG and QUE, respectively. The figure was produced using the ESPript server (http://espript.ibcp.fr/ESPript/ESPript/) [38].

To identify the catalytic residues, SbFLS was homologously modeled on the 1.75 Å resolution structure of *A. thaliana *anthocyanidin synthase (AtANS; PDB 1GP6) [25]. The overall structure of SbFLS and AtANS is very similar, with a *β* jelly-roll fold surrounded by tens of short and long *α*-helices (Figure 3(a)). The iron metal may be ligated with a bidentate group of cosubstrate 2OG as well as 3 side-chain residues, that is, H222, D224, and H278, which are conserved in AtANS and SbFLS (Figures 3(a) and 3(b)) [15, 25]. Hydrogen bonds may form between the other side of 2OG and 3 conserved residues, that is, Y207, R288, and S290 (Figure 3(b)) [15, 25, 26]. A substrate quercetin (QUE) might be located near an iron metal ion, in which 5 invariant residues H133, F135, K203, F294, and E296 may contribute to binding stability by forming a *π*-stacking arrangement (especially F294) with the QUE ring and by hydrogen bonding. All interactions as shown here were less than 3.5 Å distant. Kinetic analyses of AtANS revealed that most of the highly conserved residues play key roles in substrate/cosubstrate binding and enzymatic catalysis through metal coordination [27].

### 3.3. Transcript Levels of SbFLS in Different Tissues

**Figure 4:**
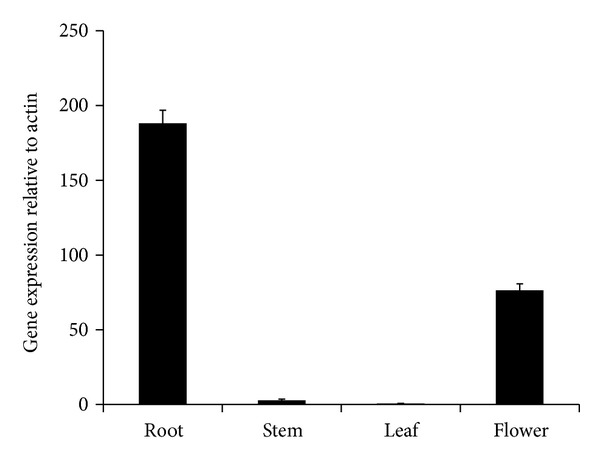
Transcript levels of *SbFLS* in different organs. The height of each bar and error bars represent means and standard errors, respectively, from 3 independent measurements.

Expression of *SbFLS *was investigated in the flowers, leaves, stems, and roots of *S. baicalensis* by qRT-PCR (Figure 4). Transcription of *SbFLS *was highest in the roots and lowest in the leaves. *SbFLS *transcription in roots was 376-, 70-, and 2.5-fold higher than in leaves, stems, and flowers. Very recently, Xu et al. [16] reported that the highest level of *G. biloba FLS *occurs in mature leaves, whereas the lowest level was observed in the roots. Expression of *AtFLS* isoforms follows different patterns in different tissues [23]. The transcription of *AtFLS1 *was highest in floral buds, flowers, and siliques, whereas the roots and shoots of young seedlings and the leaves of later vegetative stages exhibited lower expression. Other *AtFLS* isoforms were expressed at much lower levels or were undetectable in all tissues [23]. Therefore, Owens et al. [23] suggested that only AtFLS1 contributes to flavonol synthesis in *Arabidopsis*. Transcription of *Citrus unshiu* FLS was higher in young leaves than in old leaves and increased in the peel during fruit maturation [28]. Based on the transcription patterns in several plants, we conclude that FLS is differentially regulated in different plants and plant tissues.

### 3.4. Accumulation of Flavonol in Different Tissues

**Figure 5:**
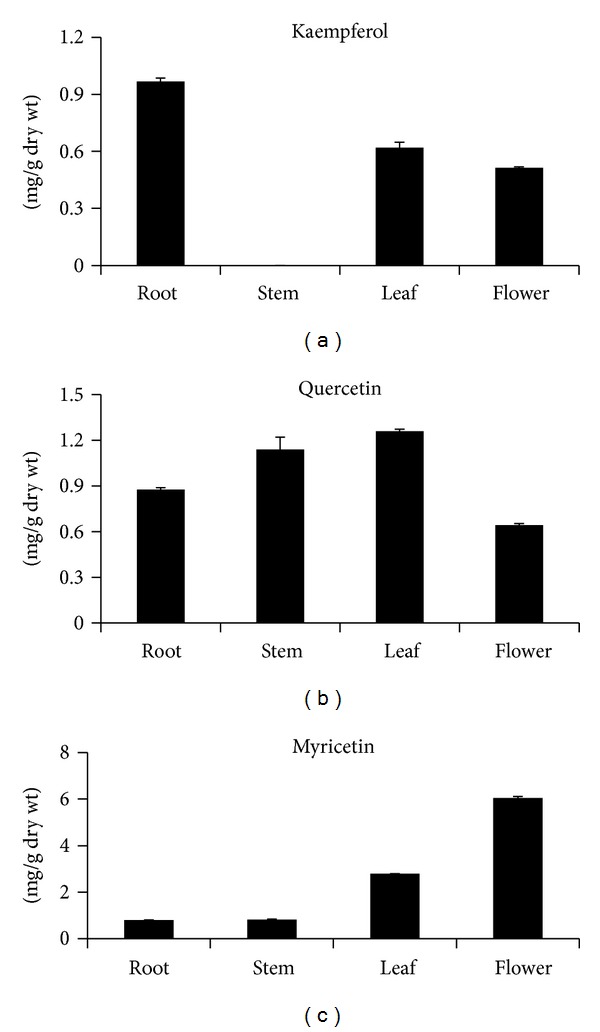
Flavonol content in different organs of *S. baicalensis. *The height of each bar and error bars represent the means and standard errors, respectively, from 3 independent measurements.

Kaempferol, quercetin, and myricetin were detected in almost all tissues (i.e., flowers, stems, leaves, and roots) by HPLC (Figure 5). The myricetin content was significantly higher than that of kaempferol and quercetin. The transcription pattern of *SbFLS* was similar to the accumulation pattern of kaempferol. Roots showed the highest kaempferol content (0.97 mg g^−1^ dry weight (DW)); the leaves and flowers contained similar amounts (0.62 and 0.51 mg g^−1^ DW), but no kaempferol was detected in the stem. Kaempferol and some glycoside derivatives have a wide range of pharmacological activities [29] and account for the utility of dry *Scutellaria *root as a multipurpose herb in oriental medicine. The leaves contained the highest levels of quercetin (1.26 mg g^−1^ DW), whereas the lowest amount of quercetin was in the flowers (0.64 mg g^−1^ DW). Interestingly, roots displayed the lowest amount of myricetin (0.8 mg g^−1^ DW), whereas the highest myricetin content was found in the flowers (6.05 mg g^−1^ DW). The myricetin content in the flowers was 2.2- and 7.5-fold higher than that in the leaves and roots, respectively. Feng and Liu [30] reported that the quercetin content of leaves was significantly higher than the content in pseudostems in different tissues of Welsh onion (*Allium fistulosum *L.).

Quercetin, kaempferol, morin, rutin, and myricetin act as antioxidants and possess anti-inflammatory, antiallergic, antiviral, and anticancer properties [31]. Quercetin is a free radical scavenger that protects against liver reperfusion ischemic tissue damage [32]. The scavenging activity of flavonols decreases as follows: myricetin > quercetin > kaempferol [33].

*SbFLS *transcription was the highest in the roots, correlating with kaempferol content in this organ. Thus, *SbFLS* might regulate the biosynthesis of kaempferol in *S. baicalensis.* Unlike kaempferol, quercetin and myricetin were most highly concentrated in the leaves and flowers, respectively. The expression level of SbFLS was not consistent with the contents of myricetin and quercetin. Owens and his colleagues [23] reported that *Arabidopsis* uses different isoforms of FLS with different substrate specificities to mediate the production of the quercetin and kaempferol in different tissue or cell types. In addition, Lillo et al. [34] described that FLS activity of the ANS enzyme may contribute to the differential accumulation of kaempferol and quercetin. Moreover, it was pointed out that differential expression of the F3′H enzyme could also mediate kaempferol and quercetin ratio in petunia flowers [35]. Stracke et al. [36] reported that FLS1 could be activated by flavonol-specific transcription factors (TFs) MYB11, MYB12, and MYB111 of *A. thaliana* and these TFs caused different spatial accumulation of specific flavonol derivatives in leaves, stems, inflorescences, siliques, and roots [23]. Several isoforms of FLS have been isolated in other plants such as *Arabidopsis* [23] and maize [24]. Therefore, we presume that SbFLS isoforms, SbANS, and SbF3′H may be contributed to the biosynthesis of quercetin and myricetin.

## 4. Conclusion

We have described the molecular cloning and characterization of a *S. baicalensis* gene encoding SbFLS. Our results suggest that SbFLS is a key potential target for the flux of the flavonol biosynthesis pathway in *S. baicalensis. *To explain adequately the flavonol biosynthesis mechanisms in *S. baicalensis*, *in vitro* enzyme assay of SbFLS isoforms, SbANS, and SbF3′H should be examined in the near future. Our study may help to determine the role of FLS and facilitate metabolic engineering of flavonol biosynthesis in *S. baicalensis.*

## Supplementary Material

Alignment of the deduced SbFLS sequence with other plant sequences. GenBank accession numbers are as follows: NtFLS (Nicotiana tabacum, ABE28017), AmFLS (Antirrhinum majus, ABB53382), CnFLS (Camellia nitidissima, ADZ28516), StFLS (Solanum tuberosum, ACN81826), GtFLS (Gentiana triflora, BAK09226), VvFLS (Vitis vinifera, BAE75809), and SbFLS (S. baicalensis, KC404852).

## Abbreviations

HPCL: High-performance liquid chromatography
CGA: Chlorogenic acid
PAL: Phenylalanine ammonia-lyase
C4H: Cinnamic acid 4-hydroxylase
4CL: 4-coumaroyl:CoA-ligase
CHI: Chalcone isomerase
F3H: Flavone 3-hydroxylase
F3′H: Flavonoid 3′-hydroxylase
F3′5′H: Flavonoid 3′5′-hydroxylase
FLS: Flavonol synthase
ANS: Anthocyanidin synthase.
