# Supplementary material for: Cloning and Characterization of a Flavonol Synthase Gene from Scutellaria baicalensis
Source: ScientificWorldJournal. 2014 Jan 28;2014:980740. doi: 10.1155/2014/980740 (PMC3927949; doi:10.1155/2014/980740)
Supplement: Supplementary file 1 — Alignment of the deduced SbFLS sequence with other plant sequences. GenBank accession numbers are as follows: NtFLS (Nicotiana tabacum, ABE28017), AmFLS (Antirrhinum majus, ABB53382), CnFLS (Camellia nitidissima, ADZ28516), StFLS (Solanum tuberosum, ACN81826), GtFLS (Gentiana triflora, BAK09226), VvFLS (Vitis vinifera, BAE75809), and SbFLS (S. baicalensis, KC404852). [file 980740.f1.docx]

**Supplementary Data**


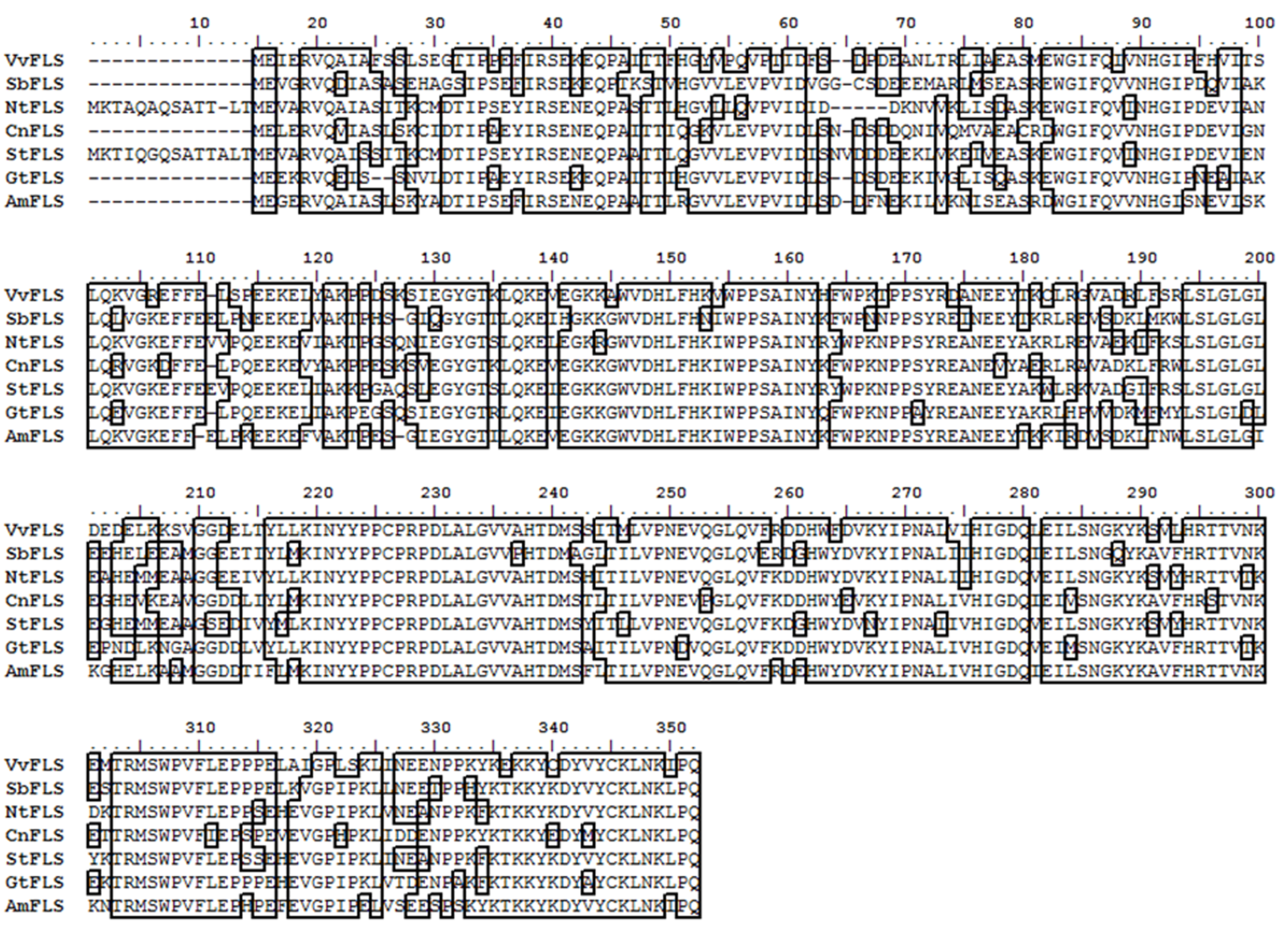


FIGURE S1: Alignment of the deduced SbFLS sequence with other plant sequences. GenBank accession numbers are as follows: NtFLS (*Nicotiana tabacum*, ABE28017), AmFLS (*Antirrhinum majus*, ABB53382), CnFLS (*Camellia nitidissima*, ADZ28516), StFLS (*Solanum tuberosum*, ACN81826), GtFLS (*Gentiana triflora*, BAK09226), VvFLS (*Vitis vinifera*, BAE75809), and SbFLS (*S. baicalensis,* KC404852).
